# Supplementary material for: A Dual Adjuvant System for Intranasal Boosting of Local and Systemic Immunity for Influenza Vaccination
Source: Vaccines (Basel). 2022 Oct 11;10(10):1694. doi: 10.3390/vaccines10101694 (PMC9611830; doi:10.3390/vaccines10101694)
Supplement: Supplementary file 1 [file vaccines-10-01694-s001.zip › vaccines-1920431-supplementary.pdf]

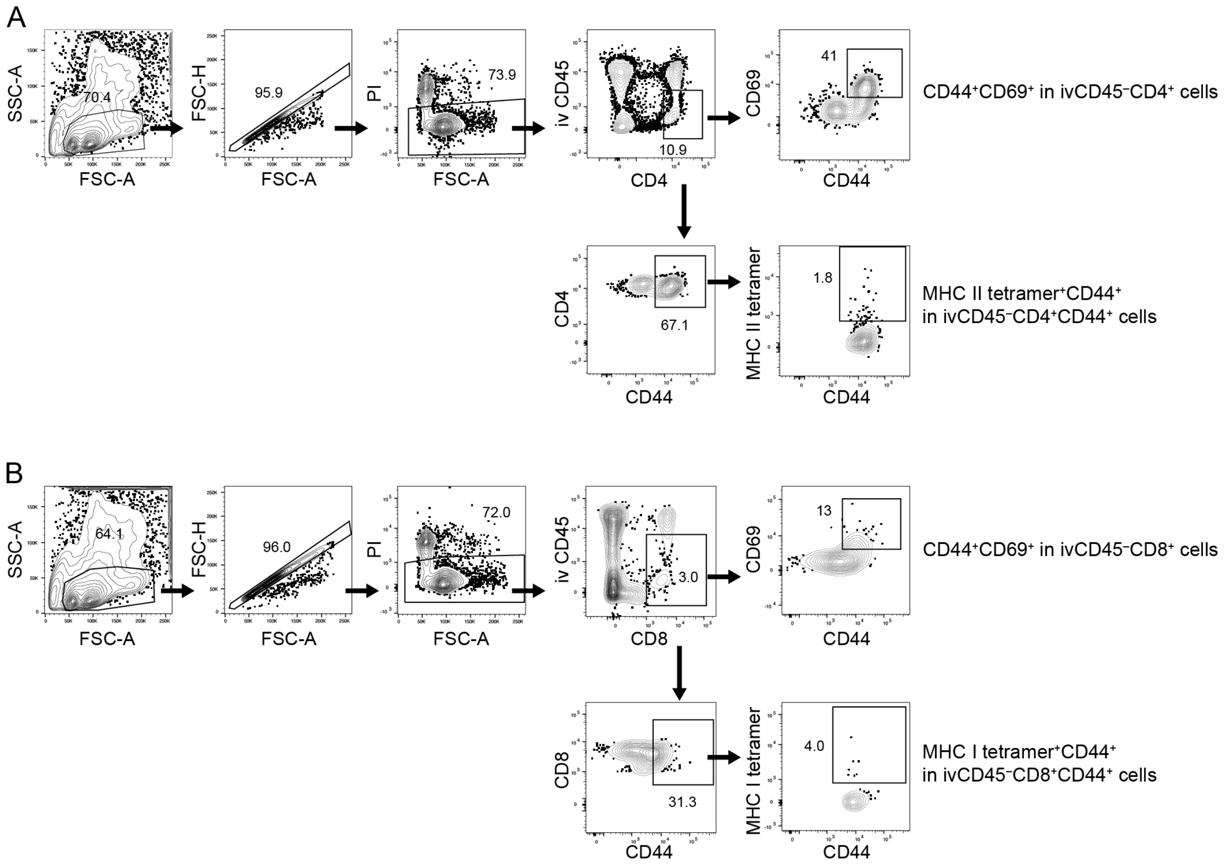

Figure S1. Gating strategies for T cell subsets in the lung. Non-circulating (tissue-resident) ivCD45^{-} CD4^{+} T cells (**A**) and ivCD45^{-}CD8^{+} T cells (**B**) in the lung were analyzed by flow cytometry. CD69^{+}CD44^{+} T cells, MHC class II or MHC class I tetramer^{+} memory T cells were subsequently identified. Antibodies and reagents used in this assay are described in Table S3.

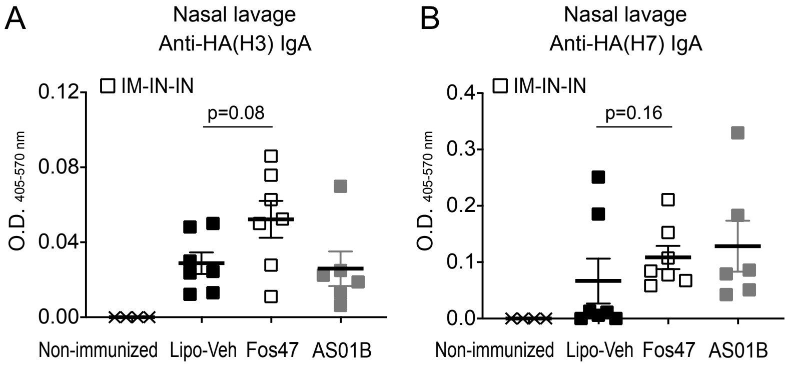

Figure S2. Cross-reactive IgA in nasal lavage induced by IN boosting with Fos47. Cross-reactivity against antigenically distinct heterologous virus strains, A/Victoria/3/1975(H3N2) and A/Netherlands/219/2003(H7N7) were tested in this assay. Female BALB/c (n=6-7/group) mice were vaccinated with IIAV plus Lipo-Veh, Fos47 or AS01B by IM-IN-IN regimen. Nasal lavage and BALF were collected on day 35. Non-immunized mice served as a negative control. IgA binding to recombinant HA protein of A/Victoria/3/1975(H3N2), or A/Netherlands/219/2003(H7N7) were evaluated by ELISA. (**A**) Anti-HA(H3) IgA in nasal lavage. (**B**) Anti-HA(H7) IgA in nasal lavage. Bars indicate means ± SEM. *P*-values are presented. Two-tailed Mann-Whitney *U*-test to compare IN-Lipo-Veh and IN-Fos47.

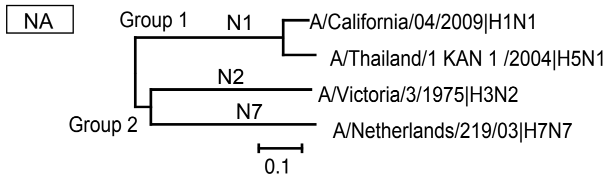

Figure S3. Phylogenetic relationship of NA proteins used in the cross-reactivity study (Figure 5). Amino acid sequences of proteins used in this study were aligned by the MUSCLE algorithm using the Influenza Research Database. Phylogenetic trees were constructed by the neighbor-joining method using MEGAX software. A/California/04/2009(H1N1pdm09) was used for vaccination. Cross-reactivity against N7 of A/Netherlands/219/2003(H7N7) were evaluated by ELISA. A/Thailand/1 KAN 1/2001(H5N1) and A/Victoria/3/1975(H3N2) are shown as a reference.

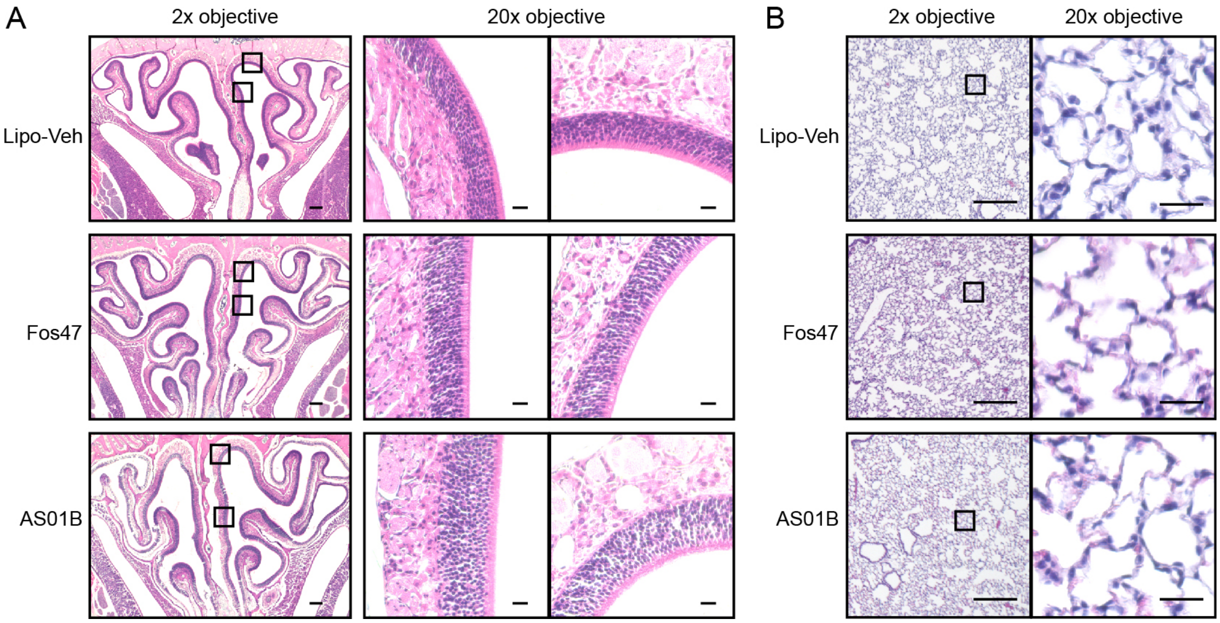

Figure S4. H&E staining of nostril and lung sections. Female BALB/c mice (n=3/group) were IN administered with 25 μL Lipo-Veh, Fos47 or AS01B. Head/nose and lung samples were collected at 24 h after IN administration. (**A** and **B**) Histologic analysis of nasal cavity (**A**) and lungs (**B**). Paraffin cross-section of lungs (5 μm thickness) were stained with HE. Low and high magnification images were obtained using 2× and 20× objective lenses, respectively. Scale bars indicates 200 μm and 20 μm in 2× objective and 20× objective, respectively.

Table S1. Reagents used in ELISA for IgG and IgA

Reagents		Source	Catalog #
Proteins for coating	*Concentrations*		
Influenza A H1N1 (A/California/04/2009) Hemagglutinin / HA Protein (His Tag)	100 ng/mL for IgG<br>1 μg/mL for IgA	Sino Biological	11055-V08B
Influenza A H1N1 (A/Puerto Rico/8/1934) Hemagglutinin / HA Protein (His Tag)	100 ng/mL for IgG<br>1 μg/mL for IgA	Sino Biological	11684-V08B
Influenza A H7N7 (A/Netherlands/219/2003) Hemagglutinin / HA Protein (His Tag)	100 ng/mL for IgG<br>1 μg/mL for IgA	Sino Biological	11082-V08B
Influenza A H3N2 (A/Victoria/3/1975) Hemagglutinin / HA1 Protein (His Tag)	1 μg/mL for IgA	Sino Biological	40396-V08H1
Influenza A H1N1 (A/California/04/2009) Neuraminidase / NA (His Tag)	100 ng/mL for IgG	Sino Biological	11058-V07B
Influenza A H7N7 (A/Netherlands/219/2003) Neuraminidase / NA Protein (His Tag)	100 ng/mL for IgG	Sino Biological	40202-V07H
Antibodies	*Dilution factor*		
IgG1-AP goat anti-mouse	1000	Southern Biotech	1070-04
IgG2a-AP goat anti-mouse	1000	Southern Biotech	1080-04
IgG-AP goat anti-mouse	1000	Southern Biotech	1030-04
IgA-AP goat anti-mouse	500	Southern Biotech	1040-04
p-Nitrophenyl Phosphate tablets (pNPP)		Sigma	N2770

Table S2. Sample dilutions for IgG and IgA evaluation

Sample types	Sample dilutions
**IgG and IgA against homologous strain**	
Serum for IgG	4-fold serial dilution (1:50 to 1:204800)
Serum for IgA	3-fold serial dilution (1:20 to 1:180)
BALF	1:10
Nasal wash	1:10
**IgG and IgA against heterologous strains**	
Serum for IgG	4-fold serial dilution (1:50 to 1:204800)
BALF	1:10 for H1RP8 and H7<br>1:2 for H3
Nasal wash	1:2

Table S3. Antibodies used in flow cytometric analysis

Antibodies (clone)	Dilution Factor	Source	Catalog #
Anti-CD4, APC/Cy7 (RM4.5)	300	BD Biosciences	565650
Anti-CD8, APC/Cy7 (53-6.7)	300	BD Biosciences	557654
Anti-CD44, FITC (IM7)	1200	BD Biosciences	553133
Anti-CD69, APC (H1.2F3)	100	eBiosciences	17-0691-82
Anti-CD16/32 (2.4G2)	300	BD Biosciences	553142
**Other reagents**			
BD Stain Buffer		BD Biosciences	554657
Propidium Iodide Staining Solution	400	BD Biosciences	556463
Class I MHC [H-2K(d)] tetramer, IYSTVASSL (533-541), PE	200	NIH tetramer core facility	
Class II MHC [I-E(d)] tetramer, SFERFEIFPKE (127-137), PE	100	NIH tetramer core facility	
***In vivo* labeling**			
Anti-CD45, PE/Cy7 (30-F11)	2.4 μg/ injection	BioLegend	103114

Table S4. Reagents used in ELISA

Reagents	Dilution Factor	Source	Catalog #
Purified rat anti-mouse IL-5	500	BD Biosciences	554393
Biotin rat anti-mouse IL-5	1000	BD Biosciences	554379
Streptavidin-HRP	1000	Thermo Fisher Scientific	434323
KPL SureBlue^{TM} TMB Peroxidase Substrate		Seracare	5120-0077
Mouse IFN-gamma DuoSet		R&D Systems	DY485
Mouse IL-17 DuoSet		R&D Systems	DY142

Table S5. Complete blood counts on days 1 and 7

Day 1	PBS	Lipo-Veh	Fos47	AS01B
**Parameters (units)**	Mean ± SEM	Mean ± SEM	Mean ± SEM	Mean ± SEM
White blood cells (× 10^3/μL)	8.4 ± 0.8	8.6 ± 0.4	9.5 ± 0.7	8.7 ± 0.4
Segmented neutrophils # (× 10^3/μL)	3.4 ± 0.3	3.3 ± 0.2	3.5 ± 0.2	3.6 ± 0.2
Lymphocytes # (×10^3/μL)	0.6 ± 0.1	0.7 ± 0.1	0.8 ± 0.1	0.6 ± 0.1
Monocytes # (× 10^3/μL)	2.9 ± 0.3	3.3 ± 0.2	3.8 ± 0.3	3.1 ± 0.2
Eosinophils # (× 10^3/μL)	1.5 ± 0.2	1.2 ± 0.2	1.3 ± 0.1	1.4 ± 0.1
Basophils # (× 10^3/μL)	0.040 ± 0.010	0.037 ± 0.009	0.057 ± 0.003	0.033 ± 0.007
Segmented neutrophils (%)	40.4 ± 2.1	38.7 ± 2.1	37.4 ± 0.4	41.0 ± 1.0
Lymphocytes (%)	6.9 ± 0.9	8.7 ± 1.2	8.7 ± 0.9	6.4 ± 1.2
Monocytes (%)	34.4 ± 2.6	38.1 ± 1.4	39.8 ± 0.9	35.9 ± 0.8
Eosinophils (%)	17.8 ± 0.5	14.2 ± 1.3	13.5 ± 0.4	16.3 ± 1.8
Basophils (%)	0.5 ± 0.1	0.4 ± 0.1	0.6 ± 0.1	0.4 ± 0.1
Red blood cells (%)	8.9 ± 0.2	9.1 ± 0.1	9.3 ± 0.1	8.8 ± 0.1
Hemoglobin (g/dL)	13.8 ± 0.4	14.5 ± 0.2	14.6 ± 0.2	14.2 ± 0.2
Hematocrit (%)	49.5 ± 0.8	50.4 ± 0.1	51.7 ± 0.2	50.1 ± 0.9
Mean corpuscular volume (fL)	56.0 ± 0.6	55.5 ± 0.8	55.9 ± 0.7	56.7 ± 0.4
Mean corpuscular hemoglobin (Pg)	15.6 ± 0.1	16.0 ± 0.1	15.8 ± 0.4	16.1 ± 0.2
Mean corpuscular hemoglobin concentration (g/dL)	27.9 ± 0.3	28.8 ± 0.3	28.3 ± 0.3	28.4 ± 0.2
Red blood cell distribution width (%)	19.8 ± 0.8	18.6 ± 0.5	19.3 ± 0.3	18.6 ± 0.1
Platelets (× 10^3/μL)	798.3 ± 8.7	774.7 ± 25.6	774.0 ± 2.0	716.0 ± 35.8
Mean platelet volume (fL)	5.2 ± 0.1	4.8 ± 0.1*	5.0 ± 0.1	5.0 ± 0.1
**Day 7**	**PBS**	**Lipo-Veh**	**Fos47**	**AS01B**
**Parameters (units)**	Mean ± SEM	Mean ± SEM	Mean ± SEM	Mean ± SEM
White blood cells (× 10^3/μL)	8.6 ± 0.6	7.9 ± 1.0	6.3 ± 0.4	8.1 ± 0.2
Segmented neutrophils # (× 10^3/μL)	3.5 ± 0.2	3.1 ± 0.3	2.7 ± 0.2	3.5 ± 0.2
Lymphocytes # (× 10^3/μL)	1.0 ± 0.1	0.8 ± 0.1	0.7 ± 0.1	0.9 ± 0.2
Monocytes # (× 10^3/μL)	2.7 ± 0.2	2.8 ± 0.4	2.0 ± 0.1	2.1 ± 0.3
Eosinophils # (× 10^3/μL)	1.4 ± 0.1	1.2 ± 0.2	0.9 ± 0.1	1.5 ± 0.1
Basophils # (× 10^3/μL)	0.053 ± 0.008	0.035 ± 0.012	0.035 ± 0.006	0.050 ± 0.012
Segmented neutrophils (%)	40.9 ± 1.2	40.1 ± 1.5	42.5 ± 0.5	43.2 ± 2.1
Lymphocytes (%)	11.0 ± 1.2	9.6 ± 0.6	11.2 ± 1.2	11.0 ± 2.2
Monocytes (%)	31.0 ± 1.0	35.3 ± 2.1	31.5 ± 1.1	26.3 ± 3.5
Eosinophils (%)	16.5 ± 0.8	14.7 ± 1.8	14.3 ± 1.7	18.9 ± 0.3
Basophils (%)	0.6 ± 0.1	0.4 ± 0.1	0.5 ± 0.1	0.6 ± 0.2
Red blood cells (%)	9.0 ± 0.3	9.2 ± 0.1	9.1 ± 0.1	9.0 ± 0.3
Hemoglobin (g/dL)	13.7 ± 0.5	14.0 ± 0.2	14.4 ± 0.1	13.9 ± 0.2
Hematocrit (%)	50.0 ± 1.4	50.7 ± 0.4	50.2 ± 1.2	49.6 ± 1.0
Mean corpuscular volume (fL)	55.7 ± 0.2	55.0 ± 0.7	55.4 ± 0.7	55.2 ± 0.7
Mean corpuscular hemoglobin (Pg)	15.3 ± 0.2	15.2 ± 0.1	15.9 ± 0.2	15.5 ± 0.3
Mean corpuscular hemoglobin concentration (g/dL)	27.4 ± 0.4	27.7 ± 0.5	28.7 ± 0.6	28.1 ± 0.3
Red blood cell distribution width (%)	19.1 ± 0.2	18.7 ± 0.5	19.1 ± 0.4	18.8 ± 0.2
Platelets (× 10^3/μL)	895.8 ± 27.7	841.3 ± 29.8	757.3 ± 91.3	872.3 ± 26.2
Mean platelet volume (fL)	5.1 ± 0.04	4.8 ± 0.07*	5.0 ± 0.13	5.1 ± 0.07

Female BALB/c mice were intranasally administered with 25 μL PBS, Lipo-Veh, Fos47 and AS01B. Whole blood was collected on days 1 or 7 after administration. N=3/group for day 1, n=4/group for day 7. *P<0.05, Kruskal-Wallis with Dunn's post hoc test to compare to PBS.

Table S6. Serum chemistry profiles on days 1 and 7

**Day 1**	**PBS**	**Lipo-Veh**	**Fos47**	**AS01B**
**Parameters (units)**	Mean ± SEM	Mean ± SEM	Mean ± SEM	Mean ± SEM
Albumin (g/dL)	3.9 ± 0.1	3.5 ± 0.1	3.5 ± 0.4	4.0 ± 0.1
Alkaline phosphatase (U/L)	164.0 ±10.4	194.7 ± 16.1	148.7 ± 22.3	180.3 ± 3.8
Alanine transaminase (U/L)	44.7 ± 1.3	42.7 ± 5.9	33.0 ± 6.4	38.0 ± 3.8
Amylase (U/L)	672.3 ± 35.6	815.3 ± 6.2	603.3 ± 127.6	706.7 ± 19.4
Bilirubin, total (mg/dL)	0.2 ± 0.00	0.2 ± 0.00	0.2 ± 0.00	0.2 ± 0.00
Blood urea nitrogen (mg/dL)	25.0 ± 0.0	26.0 ± 1.0	21.7 ± 3.5	20.0 ± 0.6
Calcium (mg/dL)	10.3 ± 0.1	10.0 ±0.1	9.0 ± 1.2	10.1 ± 0.1
Phosphorus (mg/dL)	7.5 ± 0.4	7.3 ± 0.4	5.7 ± 0.8	6.9 ± 0.1
Creatinine (mg/dL)	0.2 ± 0.03	0.4 ± 0.09	0.2 ± 0.03	0.2 ± 0.03
Glucose (mg/dL)	137.3 ± 4.7	157.0 ± 10.6	118.3 ± 4.9	132.0 ± 2.1
Sodium (mmol/L)	154.3 ± 1.8	154.0 ± 1.5	140.3 ± 15.2	154.0 ± 1.2
Potassium (mmol/L)	7.2 ± 0.1	7.1 ± 0.1	5.2 ± 1.5	7.0 ± 0.0
Total protein (g/dL)	4.7 ± 0.1	4.3 ± 0.2	4.1 ± 0.5	4.9 ± 0.1
Globulin, calculated (g/dL)	0.8 ± 0.00	0.8 ± 0.09	0.7 ± 0.12	0.9 ± 0.12
**Day 7**	**PBS**	**Lipo-Veh**	**Fos47**	**AS01B**
**Parameters (units)**	Mean ± SEM	Mean ± SEM	Mean ± SEM	Mean ± SEM
Albumin (g/dL)	4.1 ± 0.1	4.2 ± 0.0	4.2 ± 0.1	4.3 ± 0.1
Alkaline phosphatase (U/L)	156.8 ± 5.8	143.3 ± 7.7	154.7 ± 13.3	152.5 ± 3.9
Alanine transaminase (U/L)	38.0 ± 1.8	57.0 ± 7.2	38.3 ± 6.2	35.8 ± 3.3
Amylase (U/L)	627.3 ± 14.8	768.8 ± 76.0	613.0 ± 52.1	720.5 ± 93.7
Bilirubin, total (mg/dL)	0.3 ± 0.03	0.3 ± 0.03	0.3 ± 0.03	0.3 ± 0.03
Blood urea nitrogen (mg/dL)	15.5 ± 1.3	18.5 ± 1.2	16.3 ± 1.3	18.5 ± 1.0
Calcium (mg/dL)	9.7 ± 0.1	9.7 ± 0.1	9.6 ± 0.1	9.6 ± 0.1
Phosphorus (mg/dL)	6.5 ± 0.23	6.3 ± 0.03	6.0 ± 0.23	5.7 ± 0.47
Creatinine (mg/dL)	0.4 ± 0.1	0.3 ± 0.1	0.3 ± 0.1	0.2 ± 0.0
Glucose (mg/dL)	146.5 ± 5.5	141.8 ± 9.1	138.0 ± 1.5	138.8 ± 6.7
Sodium (mmol/L)	152.5 ± 0.6	153.3 ± 0.5	152.7 ± 0.9	153.5 ± 0.6
Potassium (mmol/L)	6.7 ± 0.2	7.0 ± 0.3	6.3 ± 0.2	6.7 ± 0.3
Total protein (g/dL)	4.8 ± 0.06	4.9 ± 0.04	4.8 ± 0.06	4.9 ± 0.09
Globulin, calculated (g/dL)	0.7 ± 0.04	0.7 ± 0.06	0.7 ± 0.03	0.7 ± 0.03

Female BALB/c mice were intranasally administered with 25 µL PBS, Lipo-Veh, Fos47 and AS01B. Sera were collected on days 1 or 7 after administration. N=3/group for day 1, n=4/group for day 7, there was no statistical significance compared to PBS in each time point by Kruskal-Wallis with Dunn's post hoc test.
